# An Account of Diseases in an Eastern District of London, from February 20 to March 20, 1810

**Published:** 1810-04

**Authors:** 


### *An Account of Diseases in an Eastern District of London, from February 20 to March 20,* 1810.

| | |
|---|---|
| ACUTE DISEASES. | |
| Pneumonia | 5 |
| Peripneumonia Notha | 6 |
| Rheumatismus Acutus | 4 |
| CHRONIC DISEASES. | |
| Tussis | 20 |
| Dyspnœa | 14 |
| Tussis cum Dyspnœa | 24 |
| Catarrhus | 3 |
| Phthisis Pulmonalis | 3 |
| Hydrops Pectoris | 6 |
| Pleurodyne | 5 |
| Anasarca | 4 |
| Enterodynia | 4 |
| Hypochondriasis | 3 |
| Menorrhagia | 6 |
| Fluor Albus | 9 |
| Dysuria | 5 |
| Rheumatismus Chronicus | 9 |
| PUERPERAL DISEASES. | |
| Peritonitis | 2 |
| Menorrhagia Lochialis | 4 |
| Ischuria | 3 |
| Mastodynia | 5 |
| Dolores Post Partum | 6 |
| INFANTILE DISEASES. | |
| Diarrhœa | 5 |
| Convulsio | 3 |
| Tabes Mesenterica | 4 |
| Ophthalmia | 2 |

Many of the cases of pulmonic diseases, which have lately been referred to, terminated favourably. In a few instances, instances hæmoptysis has occurred. This, though it has appeared to give some temporary relief by the abatement of pneumonic symptoms, must yet be considered as an unfavourable circumstance, as it is a strong indication, especially in younger subjects, of a predisposition to phthisis. In one instance, by taking away a little blood, and prescribing a very strict regimen, in which every thing that tended to quicken circulation was expressly forbidden, the appearance of this symptom was for the present suspended; it returned, however, in a few weeks, and, from the sensations the patient describes, it seems probable that it will soon make its appearance again, and continue by its frequent recurrence to keep up an alarm respecting its future consequences.

